# Cholestasis alters brain lipid and bile acid composition and compromises motor function in neonatal piglets

**DOI:** 10.14814/phy2.15368

**Published:** 2022-07-12

**Authors:** Nicole Lind Henriksen, Svend Høime Hansen, Matthew Domenic Lycas, Xiaoyu Pan, Thomas Eriksen, Lars Søndergaard Johansen, Richard R. Sprenger, Christer Stenby Ejsing, Douglas G. Burrin, Kerstin Skovgaard, Vibeke Brix Christensen, Thomas Thymann, Stanislava Pankratova

**Affiliations:** ^1^ Comparative Pediatrics and Nutrition, Department of Veterinary and Animal Sciences University of Copenhagen Frederiksberg C Denmark; ^2^ Department of Clinical Biochemistry Copenhagen University Hospital, Rigshospitalet Copenhagen Ø Denmark; ^3^ Department of Neuroscience University of Copenhagen Copenhagen N Denmark; ^4^ Department of Veterinary Clinical Sciences University of Copenhagen Frederiksberg C Denmark; ^5^ Department of Pediatric Surgery Copenhagen University Hospital, Rigshospitalet Copenhagen Ø Denmark; ^6^ Department of Biochemistry and Molecular Biology, VILLUM Center for Bioanalytical Sciences University of Southern Denmark Odense M Denmark; ^7^ Department of Pediatrics, United States Department of Agriculture, Agricultural Research Service Children's Nutrition Research Center, Baylor College of Medicine Houston Texas USA; ^8^ Department of Biotechnology and Biomedicine Technical University of Denmark Lyngby Denmark; ^9^ Department of Pediatrics and Adolescent Medicine Copenhagen University Hospital, Rigshospitalet Copenhagen Ø Denmark

**Keywords:** bile acids, brain, cholestasis, lipids, motor skills

## Abstract

Infants with neonatal cholestasis are prone to neurodevelopmental deficits, however, the underlying pathogenesis is unclear. Lipid malabsorption and accumulation of potentially neurotoxic molecules in the blood such as bile acids are important yet relatively unexplored pathways. Here, we developed a translational piglet model to understand how the molecular bile acid and lipid composition of the brain is affected by this disease and relates to motor function. Piglets (8‐days old) had bile duct ligation or sham surgery and were fed a formula diet for 3 weeks. Alongside sensory‐motor deficits observed in bile duct‐ligated animals, we found a shift toward a more hydrophilic and conjugated bile acid profile in the brain. Additionally, comprehensive lipidomics of the cerebellum revealed a decrease in total lipids including phosphatidylinositols and phosphatidylserines and increases in lysophospholipid species. This was paralleled by elevated cerebellar expression of genes related to inflammation and tissue damage albeit without significant impact on the brain transcriptome. This study offers new insights into the developing brain's molecular response to neonatal cholestasis indicating that bile acids and lipids may contribute in mediating motor deficits.

## Graphical abstract

The underlying pathophysiological processes in cholestasis‐induced neurodevelopmental impairments are unclear. Using a bile duct ligation piglet model, this research demonstrates that neonatal cholestasis induces significant molecular changes in the developing brain related to bile acid and lipid composition and that these findings are paralleled by sensory‐motor deficits.

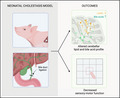

## 1 INTRODUCTION

Neonatal cholestasis (NC) is a condition where bile flow is impaired due to obstruction of bile ducts or defects in hepatocellular bile production or transport resulting in accumulation of bile constituents in the liver and blood and progressive liver damage (Götze et al., 2015). The most common and severe form of NC is biliary atresia, an obstructive, idiopathic, inflammatory, and fibrosing cholangiopathy of the biliary tree comprising around one third of all cases of NC and the leading cause of pediatric liver transplantation (Feldman & Sokol, 2013; Götze et al., 2015). However, there are several other causes of NC including a variety of genetic disorders and metabolic diseases (Götze et al., 2015). Infants with liver disease including NC are prone to hepatic encephalopathy, a neuropsychiatric manifestation of liver disease varying from attention deficits to coma (Vilstrup et al., 2014), and neurodevelopmental impairments (Rodijk et al., 2018). Recent studies have shown that children with biliary atresia achieve lower scores in cognitive, behavioral and particularly motor functional tests compared to heathy peers at 1–2 years of age as well as at school age, and that this is irrespective of liver transplant status (Ng et al., 2018; Rodijk et al., 2020). Liver‐transplanted NC patients also have poorer cognitive outcomes compared to children with stable chronic liver disease and those transplanted due to acute liver failure (Talcott et al., 2019).

Although the exact pathophysiology of hepatic encephalopathy is unclear, inflammation and neurotoxins such as ammonia are thought to play a central role (Rose et al., 2020). Studies suggest that bile acids and bile acid‐mediated signaling may also be involved by inducing blood–brain barrier permeability (Quinn et al., 2014), neuroinflammation (McMillin et al., 2017) and worsening neurological decline (McMillin et al., 2016), as described in a recent review (DeMorrow, 2019). Besides the liver‐brain axis interaction, malabsorption of nutrients, especially lipids known to be important for brain development e.g., polyunsaturated fatty acids (Socha et al., 1998), may also contribute to neurodevelopmental impairments in patients with obstructive NC. Indeed, associations between dietary lipids, brain lipid concentrations and behavioral outcomes have been demonstrated in healthy rats (Moriguchi et al., 2000).

NC is a rare disease affecting approximately 1 in 2500 infants (Mckiernan, 2002). Animal models are therefore especially important for translational research as the opportunity for clinical studies is limited. Several animal models of cholestasis exist including viral infections, chemical induction, genetic manipulation, and bile duct ligation that mimic some of the etiologies of cholestasis (Mariotti et al., 2018); however, few are based on neonatal animals and focus on neurodevelopment. Pigs were chosen for this study as they display anatomical similarities in their hepatobiliary system to humans (Ntonas et al., 2020). Furthermore, compared to rodents, pigs are more similar to humans in terms of brain development due to their gyrencephalic anatomy, white to gray matter ratio and brain growth trajectories (Duhaime, 2006; Kinder et al., 2019), and their precocial nature allows behavioral tests to be conducted at a central point in brain development (Elmore et al., 2012).

This study aimed to characterize motor function as well as brain lipid and bile acid profiles in a newly established piglet model of obstructive NC. We hypothesized that elevated plasma bile acids and lipid malabsorption would result in compositional changes in the brain that associate with motor functional impairments of NC.

## 2 MATERIALS AND METHODS

### 2.1 Animal procedures

Twenty‐four 3‐day old piglets (Danish landrace/Yorkshire/Duroc crossbreed, 12 males and 12 females) obtained from a commercial farm were stratified into two groups by body weight and sex and randomly assigned to a bile duct‐ligated (BDL, *n* = 13) or sham‐operated group (SHAM, *n* = 11). Piglets were randomly assigned to individual cages containing a heat source, soft mat, and objects for enrichment and were acclimatized for 5 days. On day 6, piglets underwent either bile duct ligation or sham surgery. Piglets were anesthetized with a 7 mg/kg intramuscular injection of a combination of tiletamine‐zolazepam (Zoletil 50 Vet; Virbac), butorphanol (Morphasol Vet; Orion Pharma A/S), ketamine (Ketaminol Vet; MSD Animal Health), and xyaline (Xysol Vet; ScanVet Animal Health A/S). To prevent surgical site infections, prophylactic antibiotics were administered intramuscularly (2.5 mg/kg Enrofloxacin; Baytril Vet, Bayer A/S). The right upper quadrant of the abdomen was aseptically prepared, and local intramuscular infiltration of lidocaine (Lidor Vet, Salfarm Denmark A/S) was administered at the surgical site. For bile duct ligation surgery, an approximately 4 cm right subcostal incision was made in the skin, underlying muscle tissue, and peritoneum. A ligature was placed on the distal part of the common bile duct close to the duodenum, and the incision was closed in two layers. Sham surgery was performed in the same way without ligation. Post‐operative analgesia consisted of an intramuscular injection of 10 μg/kg buprenorphine (Vetergesic Vet, Orion Pharma A/S) and oral administration of 30 mg/kg paracetamol (Panodil Junior 10–30 kg; GSK) for 3 days.

Piglets were weighed and their clinical status was scored daily from 1 to 4 as previously described (Holme Nielsen et al., 2019). Clinical signs associated with cholestasis (steatorrhea, dark urine, pruritus, icterus) were recorded. Throughout the study, piglets were fed increasing amounts of a milk formula diet (160–260 ml/kg/day) every 2 h (Table S1). Piglets were euthanized during the study period if humane endpoints (loss of >20% body weight, lack of pain relief, surgical complications or sepsis symptoms) were met. Piglets were euthanized on day 26, 3 weeks after surgery, by intracardic injection of pentobarbital (Euthanimal, ScanVet Animal Health A/S), and blood, liver and cerebellum tissue samples were collected.

Two piglets, one from each group, were euthanized on day 6 and 8 due to persistent lameness and an incisional incarcerated hernia. These animals were not included in statistical analyses. In one BDL piglet, the ligature was not tightened properly, and it did not develop any clinical, paraclinical or pathological signs of cholestasis and was therefore included in the SHAM group. Group sizes were, therefore, BDL *n* = 11 and SHAM *n* = 11.

### 2.2 Liver histopathology and biochemical analysis

Blood was collected from piglets on day 5 and 26 by jugular and cardiac puncture, respectively. Samples were centrifuged (2500 g, 4°C, 10 min), and plasma was collected for biochemical analysis (ADVIA 1800 Chemistry System; Siemens Healthineers AG).

Liver samples from the border of the left medial lobe were collected on day 26 and fixed in 10% neutral buffered formalin. Fixed tissue was dehydrated in graded concentrations of alcohol, cleared in xylene and embedded in paraffin (Jensen et al., 2010; Survarna et al., 2018). Tissue blocks were cut into 4–5 μm thick sections and stained with masson's trichrome and hematoxylin–eosin (Survarna et al., 2018). Additionally, immunostaining of bile duct epithelial cells using an antibody toward cytokeratin (M3515 clone AE1/AE3; Dako) was performed as previously described (Jensen et al., 2010). Antigen retrieval was in this study carried out with citrate buffer pH 6 for 2 × 5 min. Hematoxylin–eosin stained sections were used to describe liver pathomorphology, whilst masson's trichrome and cytokeratin‐stained sections were used to quantify fibrosis and bile duct proliferation, respectively, in a blinded fashion. The percentage of collagen fibers and bile duct epithelial cells in five randomly selected ×10 magnification fields were quantified using Fiji (Schindelin et al., 2012) and averaged.

### 2.3 Motor function tests

Motor function and exploration were assessed on days 15 and 25 corresponding to 10 and 20 days post‐surgery, respectively. First, piglets completed a balance beam test, as described by Roelofs et al. (Roelofs et al., 2019). Briefly, piglets completed three consecutive runs along a 180 cm long and 4.5 cm high wooden balance beam during which the number of missteps (foot placed beside beam) was registered by two observers. A wooden and plexiglass wall were placed at a distance of 2.5 cm on either side of the beam, and the beam was covered by a slip‐resistant surface. The width of the beam was adjusted according to the size of piglets (approximately 4 cm wider than the shoulder width) in order to make the test equally challenging to all animals. As the number of missteps is considered an objective parameter, observers were not blinded to treatment groups (Roelofs et al., 2019). One piglet from the SHAM group was excluded from the analysis on day 25 due to continuous off‐task behavior.

Secondly, piglets were placed in a 2 × 2 m open field arena made up of wooden walls and black rubber flooring for 5 min. During the first 2 min, a birds‐eye‐view open field video recording was made to assess locomotion and exploration. Tracking analysis of open field recordings was conducted using the Ethovision XT10 software (Noldus Information Technology) which generated measurements of distance traveled, velocity, time spent in different zones of the arena and number of zone transitions. During the last 3 min, piglets were recorded by a video camera mounted outside the open field arena. Using these video recordings, two independent observers scored the following parameters for gait and posture: head tilt/turn, lameness, wide/narrow stance, asymmetric body posture, muscle atrophy, involuntary muscle contractions, ataxia, dysmetria, walking into walls, stumbling/knuckling/falling and stiff gait. Each parameter was scored as 0 = absent, 1 = intermediate or 2 = clearly present. Additionally, parameters that were countable e.g., number of falls, were given 1 point each time they occurred. Results were pooled to provide a collective neuromotor score for each piglet. Observers had experience evaluating gait and posture of pigs and were blinded to treatment groups. Due to technical difficulties, two piglets, one from each group, were excluded from the neuromotor score analysis.

### 2.4 Gait analysis

Video recordings from the balance beam test were used for the gait analysis (Video S1A,B). Tracking of piglets was performed using DeepLabCut version 2.0.7.2, as described by Nath et al. (Nath et al., 2019). Training targets were selected from approximately 1000 frames. This model was trained for over 800,000 iterations utilizing a NVIDIA Quadro P5000 graphics card. The model was evaluated and demonstrated an error of the training data set of 2.26 pixels and an error on an independent test data set of 6.85 pixels. This model was trained to identify the snout of the piglet, the front shoulder, the tail base, and each independent foot. Upon collection of all tracked files, the identified locations on the piglet were subject to an in house written python script to identify aspects of the piglet's motion. This was conducted in two broad steps, first identifying the longest period of continuous motion of the piglet, and second to identify the stride length, swing time, and stance time of each foot within this time period.

Starting with the csv file produced by DeepLabCut, the positions were filtered for those that were identified with a certainty above 0.8, and a linear interpolation was performed. Next, a median filter set for 15 frames was applied to the x pixel coordinate for the nose and the x pixel coordinate for the shoulder value. The difference in value per frame was calculated for these two filtered signals and then averaged together. This was filtered further with a median filter and a mean filter, each for 15 frames. The resulting signal was an approximation for the velocity of the piglet as a function of frame in the video. The period of longest continual movement was identified by identifying the largest peak of this velocity function. Once these frames of the video were identified, the original csv file, following the certainty threshold and interpolation, was cropped to this time window.

The stride length was obtained per foot from the period of maximum continual movement by first identifying the discrete difference of the x pixel coordinate for that foot. The local maximum values from this signal were obtained, identifying the moments when the given foot was in motion. A given individual stride distance was identified by identifying the foot position in the interval prior to the movement and the interval following the movement. This distance was converted to cm by identifying the location of the foot in the context of the plank, which has known dimensions, that it was walking on. The swing time was obtained by taking the width of each peak in the discrete difference of the x pixel coordinate for that foot. The stance time was obtained by measuring the number of frames between peaks in the discrete difference of the x pixel coordinate for that foot. These aspects were chosen based off of the work of Holme Nielsen et al. (Holme Nielsen et al., 2019) The code can be found at https://github.com/Comparative‐Pediatrics‐and‐Nutrition/.

### 2.5 High‐resolution shotgun lipidomics

Prior to euthanasia on day 26, piglets were given a standardized 15 ml/kg dietary bolus. Lipid extraction and subsequent mass spectrometry‐based lipidomics and in silico total fatty acyl analysis were carried out on snap‐frozen cerebellum tissue samples, as previously described (Almeida et al., 2015; Ejsing et al., 2006; Sprenger et al., 2021).

### 2.6 Bile acids and C4

Quantification of individual unconjugated bile acids (DCA: deoxycholic acid, CDCA: chenodeoxycholic acid, CA: cholic acid, HDCA: hyodeoxycholic acid and LCA: lithocholic acid) and their glycine (G‐) and taurine (T‐) conjugates was performed following the manufacturer's instructions (Biocrates® bile acids kit; Biocrates Life Sciences Ag Innsbruck). Additional calibrators were included to quantify hyocholic acid (HCA), T‐HCA and G‐HCA (all from Cayman Chemical Company). Quantification of the bile acid biosynthesis marker C4 (7α‐hydroxy‐4‐cholesten‐3‐one) was based on a C4 calibration curve and deuterated internal standard d_7_‐C4 (d_7_‐7α‐hydroxy‐4‐cholesten‐3‐one; both from Toronto Research Chemicals).

Plasma samples (50 μl) were precipitated with 250 μl methanol mixed with the internal standard. After vigorous shaking and centrifugation (5000 g, 4°C, 10 min), supernatants were collected and stored at −20°C. Frozen cerebellum tissue samples were crushed, weighed and an average of 245 mg tissue was mixed with the methanol and internal standard combination at 1:5 (w/v). Samples were centrifuged (5000 g, 4°C, 10 min), and the supernatants were collected for chromatographic analysis. LC–MS/MS analysis of bile acids was performed using the Acquity UPLC system connected to a TQ‐S mass spectrometer (both from Waters Corporation). The UPLC fractionation of bile acids was conducted on a HSS T3 column (100 mm × 2,1 mm; Waters Corporation), and water (A) and methanol (B) supplemented with 1.25 mM ammonium formate were used as eluents. To perform the chromatographic separation, a stepwise‐increased isocratic gradient was applied, starting with 60%A:40%B and ending at 18%A:82%B. The flow rate was 0.45 ml/min. Data collection and subsequent processing were performed with the Waters MassLynx software (Version 4.1; Waters Coporation). The lower limit of detection was 0.01 μmol/L, and samples below this number were treated as missing values. Bile acids that were detected in less than five of 11 samples in each group were excluded. Total bile acid levels were determined as the sum of all bile acids measured by LC–MS/MS.

### 2.7 FGF‐19 analysis

A commercial porcine ELISA kit (RayBiotech) was used to determine plasma fibroblast growth factor 19 (FGF‐19) concentrations as previously described (Smith et al., 2020). Values below the lower limit of detection (5 pg/mL) were replaced with 5 pg/mL.

### 2.8 RNA sequencing

Snap‐frozen cerebellum tissue samples were disrupted in liquid nitrogen using a cryogenic tissue pulverizer and subsequently homogenized in QIAzol lysis reagent (Qiagen). The RNeasy Lipid Tissue Mini Kit (Qiagen) was used for tissue extraction of total RNA according to the manufacturer's instructions, and RNA quality was measured with the Bioanalyzer 2100 (Agilent) and NanoDrop 100 Spectrophotometer (Thermo Fisher). RNA integrity numbers ranged from 5.90–8.90. RNA libraries were prepared by Novogene Europe and sequenced on the Illumina NovaSeq platform generating 150‐bp paired‐end reads. RNA sequencing data were analyzed as previously described (Pan et al., 2020) using HISAT2 as the alignment tool (Kim et al., 2015).

### 2.9 qPCR

Cerebellar expression of genes related to bile acid signaling, phosphatidylinositide metabolism, inflammation and oxidative stress/apoptosis were measured by reverse transcription quantitative polymerase chain reaction (qPCR) using porcine‐specific primers designed in Primer 3 version 0.4.0 (https://primer3.ut.ee) or Primer‐BLAST (https://www.ncbi.nlm.nih.gov/tools/primer‐blast/; Table S2). Briefly, for bile acid signaling‐related genes, cDNA was synthesized from 1 μg total RNA using the High‐Capacity cDNA Reverse Transcription Kit (Thermo Fisher). qPCR was performed using the LightCycler 480 SYBR Green I Master kit and LightCycler 480 system (both from Roche) as previously described (Li et al., 2020). Relative expression of target genes were normalized to the reference gene *HPRT1* (Table S2; Li et al., 2020). For the remaining genes, cDNA was synthesized in triplicates from 500 ng total RNA using the QuantiTect Reverse Transcription Kit (Qiagen), pre‐amplified using the TaqMan PreAmp Master Mix (Applied Biosystems) and treated with exonuclease I (New England Biolabs) as previously described (Skovgaard et al., 2013). Primer sets and pre‐amplified and exonuclease‐treated cDNA including non‐template and non‐reverse transcriptase controls were loaded on a 96.96 Dynamic Array Integrated Fluidic Circuit chip (Fluidigm) and analyzed on the BioMark real‐time PCR system (Fluidigm; Skovgaard et al., 2013). *C*_*q*_ values were obtained from the Fluidigm Real‐Time PCR Analysis software (version 4.7.1; Fluidigm) and processed in GeneEx (version 8; MultiD) by correcting for PCR efficacy, normalizing to validated reference genes (*ACTB*, *GAPDH*, *HPRT1*, *RPL13A*, *YWHAE*; Table S2) and averaging cDNA triplicates, as previously explained (Skovgaard et al., 2013). Mean values from the SHAM group were set to 1, all remaining data were scaled accordingly and expression in the BDL group was calculated relative to the SHAM (Skovgaard et al., 2013).

### 2.10 Statistics

Statistical analyses were performed in R (version 4.0.3; R Foundation for Statistical Computing). A linear mixed effects model (lme function) including treatment, gender and arrival weight as fixed effects and piglet as a random effect was used to analyze repeated measurements over time (body weight, gait, and motor function tests). Continuous data (organ weights, biochemistry, histology scores, FGF‐19, C4, bile acids, qPCR, lipidomics) were analyzed using linear regression (lm function), and RNA sequencing data were analyzed by a negative binomial generalized linear model using the DESeq2 package and the same fixed effects. Model assumptions of normality and homoscedasticity of residuals and fitted values were assessed, and data transformation was performed if necessary. A non‐parametric test (wilcox. test function) was conducted for data that were unable to be transformed properly. Lipidomics and fluidigm qPCR data were log transformed. A Benjamini‐Hochberg p value adjustment was used for RNA sequencing and lipidomics data to correct for multiple comparisons. *p* ≤ 0.05 was considered statistically significant. Data are presented as means with standard deviations (*SD*) or percentages.

## 3 RESULTS

### 3.1 Clinical findings

The growth rate was similar between groups (BDL: 42.8 ± 5.1 g/kg/day, SHAM: 42.6 ± 9.5 g/kg/day). The BDL group developed clinical signs of cholestasis including persistent steatorrhea and dark urine. Icteric coloration of the sclera was observed in six BDL piglets on day 25–26, whereas no animals developed pruritus. A distended abdomen was observed in many BDL piglets, which was accounted for by enlargement of the liver and colon. Three piglets from the SHAM group developed diarrhea between day 12–16 that required antibiotic treatment with gentamicin (Gentocin Vet, ScanVet Animal Health A/S) and amoxicillin/clavulanic acid (Bioclavid, Sandoz International GmbH). One BDL piglet had an aversive reaction to the anesthesia and had a long post‐operative recovery time.

### 3.2 Plasma biochemical profile

Biochemical analysis demonstrated increased levels of aspartate transaminase, alkaline phosphatase, gamma‐glutamyltransferase, total bilirubin, conjugated bilirubin, ammonia, triglycerides, iron, cholesterol, and potassium in the BDL relative to SHAM group on day 26 (all *p* < 0.05). On the contrary, alanine transaminase, albumin, phosphate, magnesium and total protein (all *p* ≤ 0.05) were elevated in the SHAM relative to BDL group on day 26 (Table S3).

### 3.3 Organ weights, macroscopic pathology and histopathology

**FIGURE 1:**
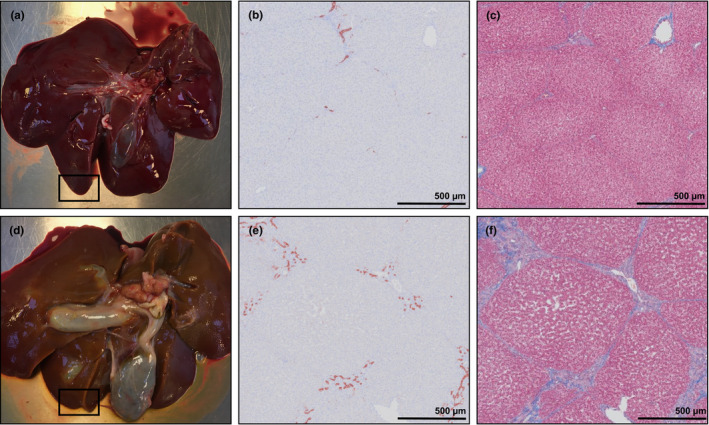
Representative images of macroscopic and histological liver pathology from the SHAM‐operated (SHAM, a–c) and bile duct‐ligated (BDL, d–f) group on day 26. Images b, c, e and f are from the highlighted area in images a and d. The left panel shows the liver of a SHAM (a) and BDL (d) piglet, where cystic dilation of the biliary tree and gall bladder are seen in image d. The middle and right panel show cytokeratin staining for bile duct epithelium and masson's trichrome staining for collagen in a SHAM (b, c) and BDL (e, f) piglet, respectively. 2× objective. Scale bars are 500 μm.

At euthanasia, the BDL group had higher relative organ weights for liver and full colon, whilst the SHAM group had higher carcass weights (all *p* < 0.05; Table S4). Absolute brain regional weights and water percentage were similar between groups (Table S5). BDL piglets had hepatomegaly and cystic dilation of the biliary tree and gall bladder (Figure 1d). Additionally, two BDL piglets developed a biliary cyst. Histological findings in the BDL group consisted of porto‐portal bridging fibrosis, bile plugs and portal bile duct proliferation consistent with histological findings in children with obstructive NC (Lee & Looi, 2009). BDL piglets had varying degrees of portal inflammatory cell infiltrates, and a single BDL piglet had hepatocellular swelling. The amount of fibrosis (BDL: 6.88 ± 2.44%, SHAM: 0.85 ± 0.30%, *p* < 0.001) and bile duct proliferation (BDL: 1.96 ± 0.71%, SHAM: 0.12 ± 0.03%, *p* < 0.001) were higher in the BDL relative to SHAM group (Figure 1).

### 3.4 Motor function tests and gait analysis

**FIGURE 2:**
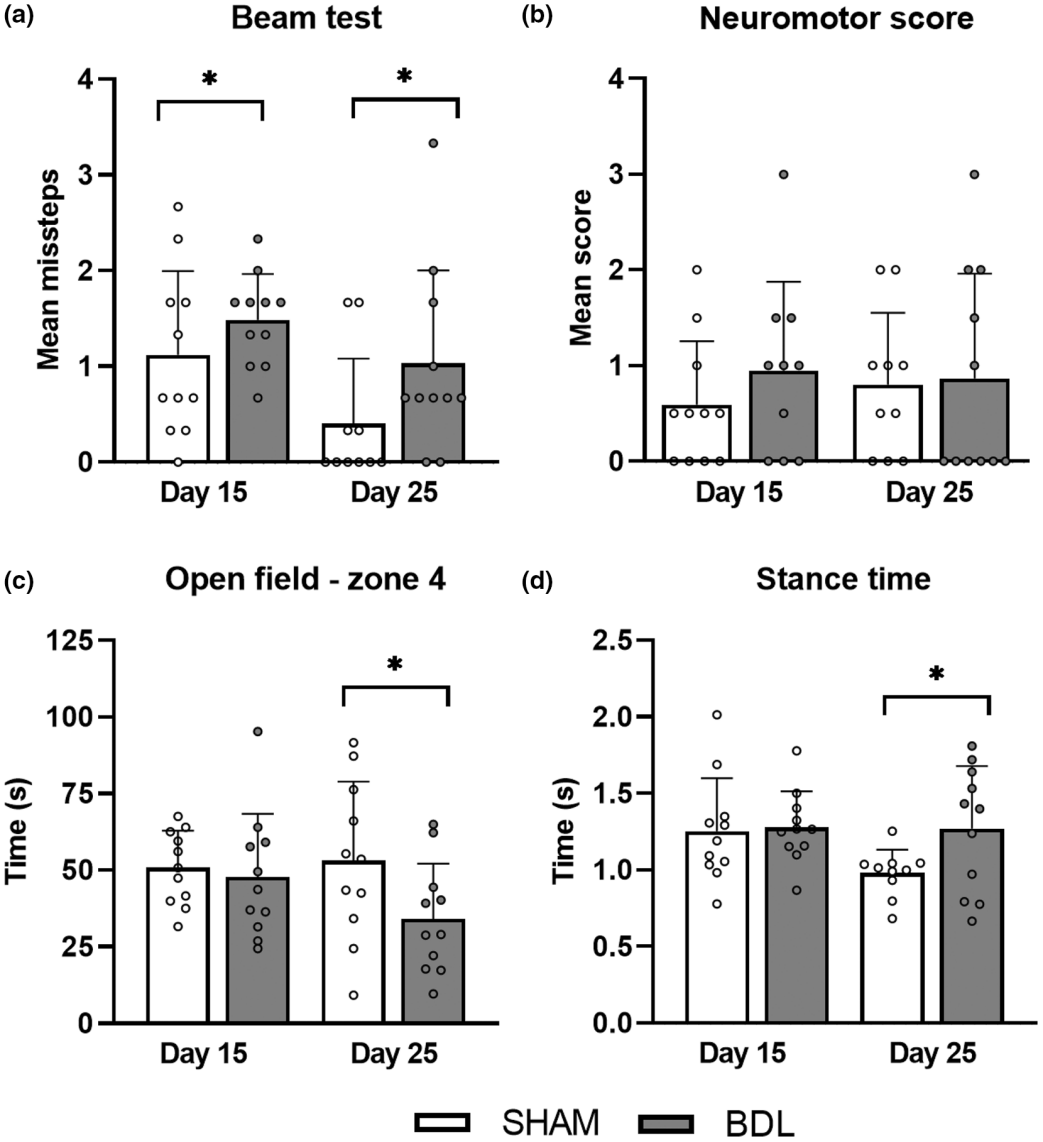
Motor function tests: (a) beam test, (b) neuromotor score, (c) open field test—time spent in zone 4 of the arena (entrance zone) and (d) absolute stance time in SHAM‐operated (SHAM, *n* = 10–11) and bile duct‐ligated (BDL, *n* = 10–11) piglets on day 15 and 25. *p < 0.05. Data are presented as means ± *SD*.

The mean number of missteps in the beam test was higher in the BDL compared to SHAM group over time (*p* < 0.05; Figure 2a). The neuromotor score was similar for both groups (Figure 2b). SHAM piglets spent more time in zone 4 of the open field arena (entrance zone) compared to BDL piglets on day 25 (*p* < 0.05; Figure 2c). There were no differences between groups for the remaining open field parameters (Table S6). Gait analysis demonstrated longer absolute and normalized stance times in BDL compared to SHAM piglets on day 25 (Figure 2d; Table S6). Stride length, speed, and swing time were similar between groups (Table S6). There were no effects of sex for any of the motor function parameters.

### 3.5 Lipidomics

**FIGURE 3:**
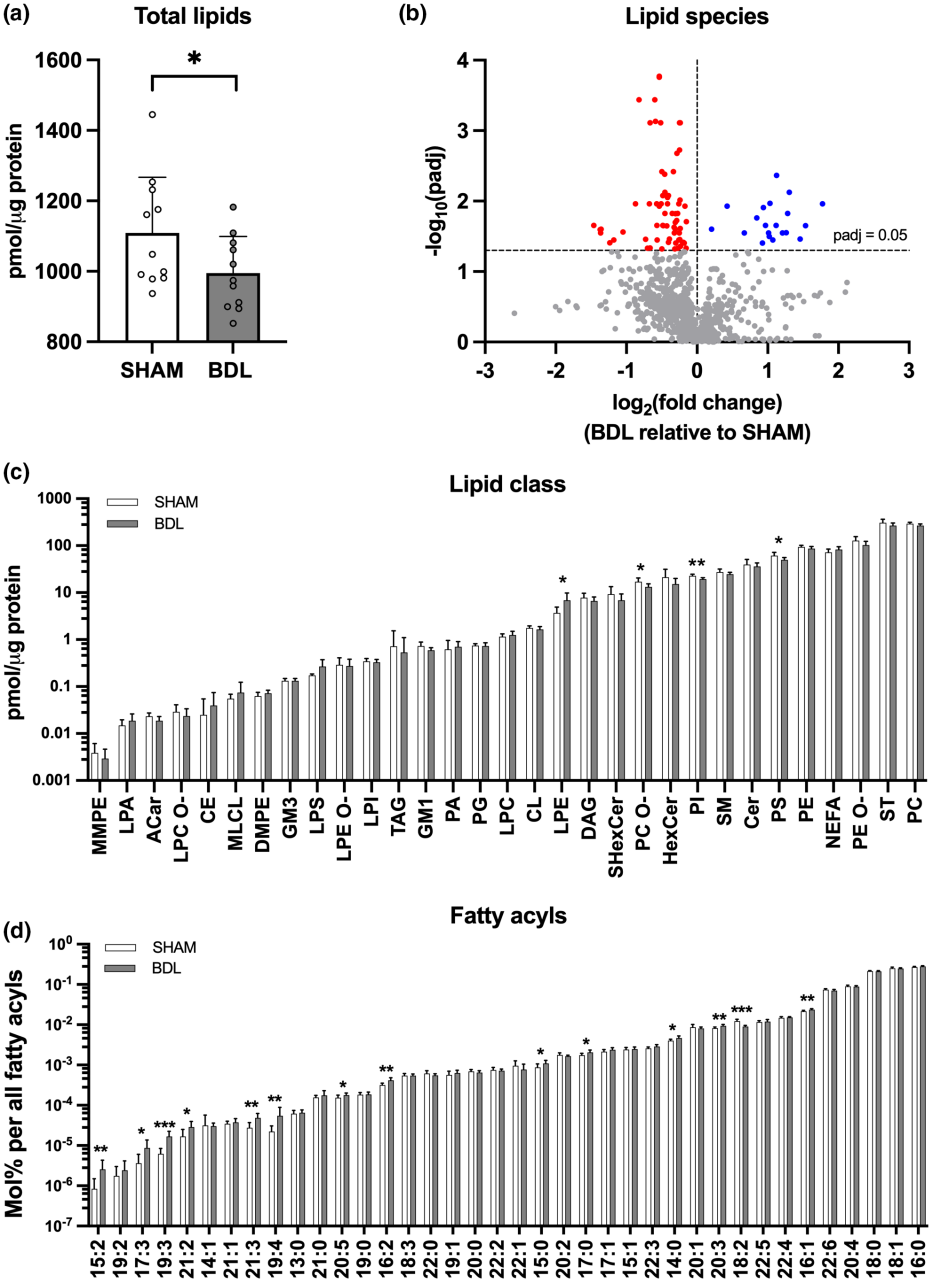
Lipid profile of the cerebellum: (a) Total lipids, (b) lipid species (c) lipid classes and (d) total fatty acyls in SHAM‐operated (SHAM, *n* = 8–11) and bile duct‐ligated (BDL, *n* = 8–11) piglets on day 26. *p < 0.05, **p < 0.01, ***p < 0.001. Benjamini‐Hochberg‐adjusted *p* values are reported in b, c, and d. Data are presented as means ± *SD* (a, c, d) or fold change of the BDL relative to SHAM group (b). ACar, acylcarnitine; CE, cholesteryl ester; Cer, ceramide; CL, cardiolipin; DAG, diacylglycerol; DMPE, dimethyl‐phosphatidylethanolamine; GM1, monosialotetrahexosylganglioside; GM3, monosialodihexosylganglioside; HexCer, hexosyl ceramide; LPA, lysophosphatidic acid; LPC, lysophosphatidylcholine; LPC O‐, lysoalkylphosphatidylcholine; LPE, lysophosphatidylethanolamine; LPE O‐, lysoalkylphosphatidylethanolamine; LPI, lysophosphatidylinositol; LPS, lysophosphatidylserine; MLCL, monolysocardiolipin; MMPE, monomethylphosphatidylethanolamine; NEFA, non‐esterified fatty acyl, phosphatic acid, phosphatidylcholine; PC O‐, alkylphosphatidylcholine; PE, phosphatidylethanolamine; PE O‐, alkylphosphatidylethanolamine; PG, phosphatidylglycerol; PI, phosphatidylinositol; PS, phosphatidylserine; SHexCer, sulfatides hexosyl ceramide; SM, sphingomyelin; ST, cholesterol; TAG, triacylglyceride.

Lipidomics analysis quantified 1085 lipid species from 31 lipid classes. The total amount of lipids in the cerebellum was higher in the SHAM compared to BDL group (*p* < 0.05; Figure 3a). At the lipid class level, phosphatidylinositol (PI), phosphatidylserine (PS) and alkylphosphatidylcholine (PC O‐) were less abundant and lysophosphatidylethanolamine (LPE) more abundant in the BDL group (all *p* < 0.05; Figure 3c). At the level of individual lipid species, 94 were present in only one of the groups and were therefore omitted from the statistical analysis (Table S7). Of the remaining monitored lipid species, 88 were differentially expressed between BDL and SHAM piglets (all *p* < 0.05; Figure 3b). The main differences were related to lower concentrations of phosphatidylcholine (PC), PC O‐, PI and PS species (~67% of downregulated lipid species) and higher concentrations of lysophosphatidylcholine (LPC) and LPE species (~60% of upregulated lipid species) in the BDL group relative to the SHAM. In silico total fatty acyl analysis revealed that BDL piglets had a higher number of lipids with 14:0, 15:0, 15:2, 16:1, 16:2, 17:0, 17:3, 19:3, 19:4, 20:3, 20:5; 21:2 and 21:3 fatty acyl chains, whereas SHAM piglets had more lipids with 18:2 fatty acyl chains (all *p* < 0.05; Figure 3d). Lipids with 17:2 fatty acyl chains were only present in the BDL group and were therefore excluded from the statistical analysis.

### 3.6 Bile acids, FGF‐19 and C4

**FIGURE 4:**
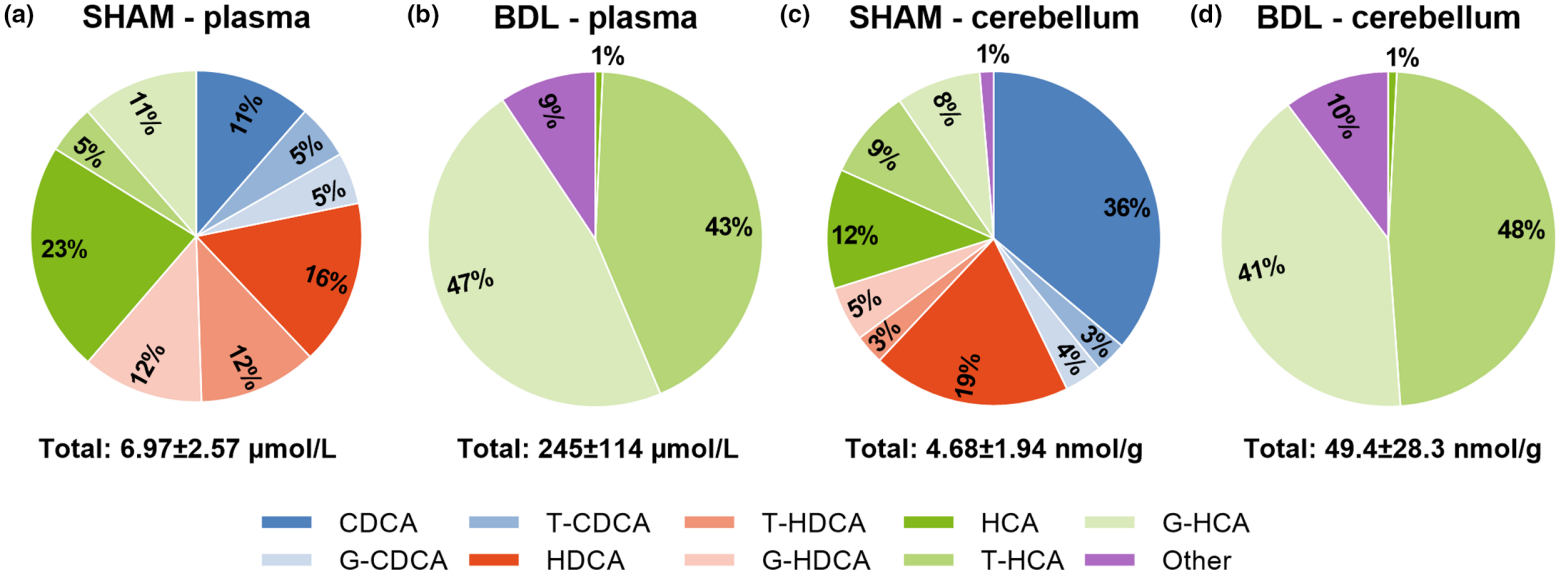
Relative bile acid levels in plasma (a, b) and cerebellum tissue (c, d) in SHAM‐operated (SHAM) and bile duct‐ligated (BDL) piglets on day 26. Data are provided as the percentage of individual bile acids out of the total amount of bile acids measured by LC–MS/MS. Low‐abundance bile acids are grouped as “other” and their absolute values can be seen in Table 1. CDCA, chenodeoxycholic acid; HCA, hyocholic acid; HDCA, hyodeoxycholic acid; G‐: glycine conjugated; T‐, taurine conjugated.

**FIGURE 5:**
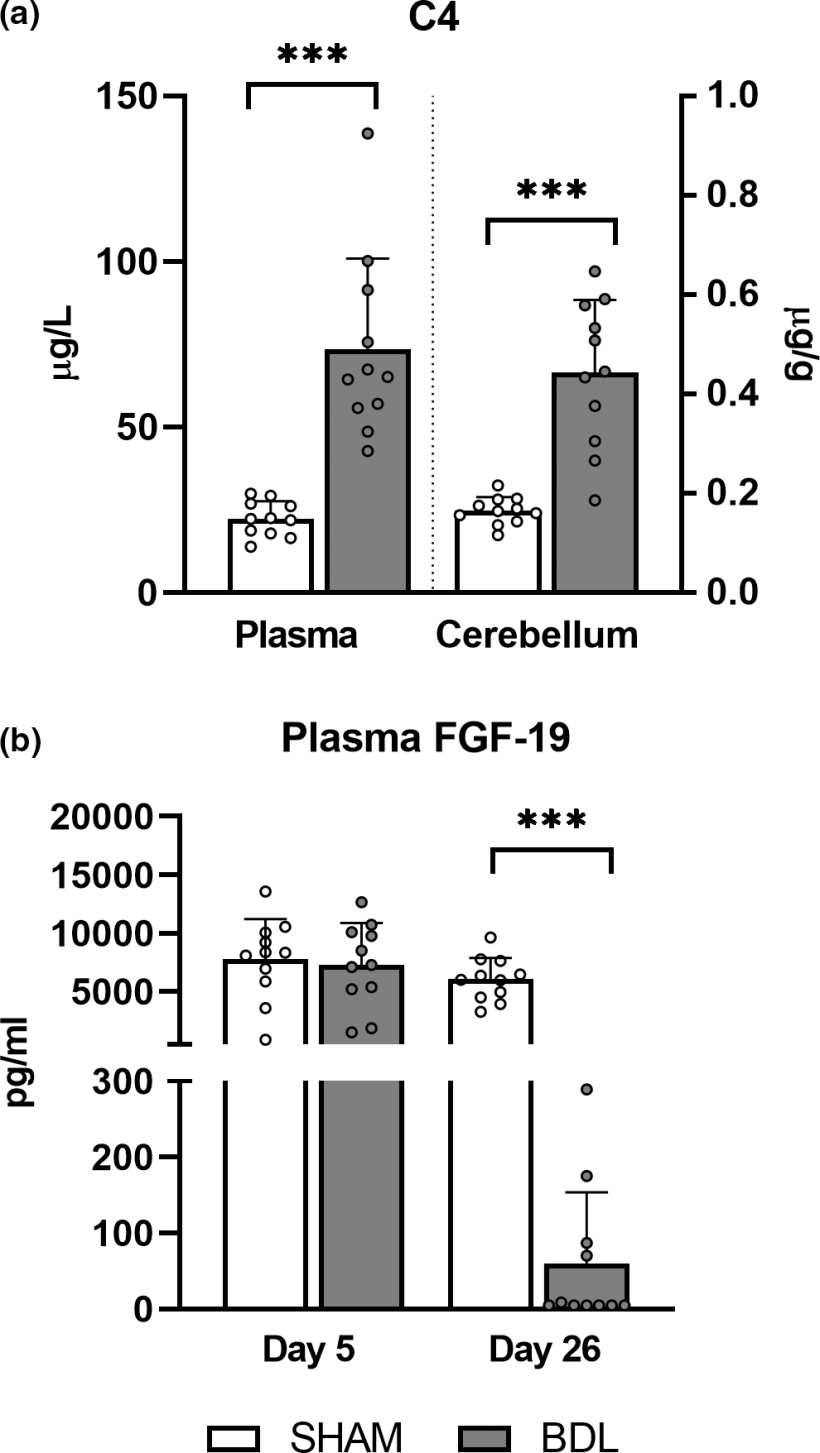
Day 26 C4 (7‐alpha‐hydroxy‐4‐cholesten‐3‐one) levels in plasma and cerebellum tissue (a), and day 5 and 26 plasma fibroblast growth factor 19 (FGF‐19) levels (b) in SHAM‐operated (SHAM, *n* = 11) and bile duct‐ligated (BDL, *n* = 11) piglets. ***p < 0.001. Data are presented as means ± *SD*.

Measurements of total bile acids on day 26 in plasma (BDL: 245 ± 114 μmol/L, SHAM: 6.97 ± 2.57 μmol/L, *p* < 0.001) and the cerebellum (BDL: 49.4 ± 28.3 nmol/g, SHAM: 4.68 ± 1.94 nmol/g, *p* < 0.001) were higher in the BDL relative to SHAM group. The bile acid profile of the BDL group was mainly composed of HCAs, whilst it was HCAs, CDCAs and HDCAs in the SHAM group (Figure 4; Table 1). The concentration of all bile acids differed between treatment groups (*p* < 0.05) with the exception of CA, HCA, and LCA in plasma and T‐HDCA in the cerebellum (Table 1). A higher percentage of bile acids were conjugated to glycine and taurine in the BDL group in both plasma (BDL: 99.2 ± 0.54%, SHAM: 51.0 ± 18.3%, *p* < 0.001) and cerebellum tissue (BDL: 98.4 ± 1.02%, SHAM: 33.3 ± 17.6%, *p* < 0.001). The primary to secondary bile acid ratio was likewise higher in the BDL group (plasma: BDL: 26.6 ± 19.2, SHAM: 1.91 ± 1.25, *p* < 0.001; cerebellum: BDL: 32.3 ± 23.6, SHAM: 3.21 ± 1.65, *p* < 0.001). C4 was elevated in both plasma and cerebellum tissue in the BDL group (both *p* < 0.001; Figure 5a), whilst plasma FGF‐19 levels were increased in the SHAM group on day 26 (*p* < 0.001; Figure 5b).

**TABLE 1:** Bile acid concentrations in plasma and cerebellum tissue on day 26 in SHAM‐operated (SHAM) and bile duct‐ligated (BDL) piglets (means ± *SD*)

Bile acid	Plasma (μmol/L)		Cerebellum (nmol/g)		p value	
	SHAM (*n* = 9–11)	BDL (*n* = 5–11)	SHAM (*n* = 6–11)	BDL (*n* = 5–11)	*p*_plasma_	*p*_cerebellum_
CA	0.02 ± 0.02	0.03 ± 0.01	0.07 ± 0.03	ND	0.2	—
G‐CA	ND	0.22 ± 0.17	ND	0.11 ± 0.09	—	—
T‐CA	ND	2.24 ± 2.01	ND	0.62 ± 0.75	—	—
LCA	0.017 ± 0.007	0.011 ± 0.003	ND	ND	0.07	—
G‐LCA	ND	ND	ND	ND	—	—
T‐LCA	ND	ND	ND	ND	—	—
CDCA	0.79 ± 0.63	0.10 ± 0.07	1.74 ± 1.40	0.14 ± 0.05	<0.001	<0.001
G‐CDCA	0.35 ± 0.13	3.36 ± 4.41	0.18 ± 0.13	0.61 ± 0.82	<0.001	<0.05
T‐CDCA	0.37 ± 0.27	4.49 ± 3.68	0.15 ± 0.15	1.47 ± 1.46	<0.001	<0.001
DCA	ND	ND	ND	ND	—	—
G‐DCA	ND	ND	ND	ND	—	—
T‐DCA	ND	ND	ND	ND	—	—
HDCA	1.12 ± 0.78	0.09 ± 0.10	0.93 ± 0.54	0.16 ± 0.06	<0.001	<0.001
G‐HDCA	0.82 ± 0.39	7.51 ± 5.83	0.25 ± 0.17	1.35 ± 1.23	<0.001	<0.001
T‐HDCA	0.80 ± 0.55	4.87 ± 3.17	0.14 ± 0.07	0.60 ± 0.55	<0.001	0.08
HCA	1.56 ± 1.06	1.75 ± 1.72	0.56 ± 0.30	0.40 ± 0.45	0.6	<0.05
G‐HCA	0.79 ± 0.62	115 ± 64.2	0.39 ± 0.26	20.3 ± 14.1	<0.001	<0.001
T‐HCA	0.33 ± 0.30	105 ± 50.6	0.42 ± 0.37	23.8 ± 14.0	<0.001	<0.001
Conjugated	3.47 ± 1.62	243 ± 113	1.43 ± 0.75	48.7 ± 28.1	<0.001	<0.001
Unconjugated	3.50 ± 2.06	1.97 ± 1.84	3.25 ± 2.01	0.69 ± 0.53	<0.05	<0.001

Abbreviations: CA, cholic acid; CDCA, chenodeoxycholic acid; DCA, deoxycholic acid; G‐, glycine conjugated; HCA, hyocholic acid; HDCA, hyodeoxycholic acid; LCA, lithocholic acid; ND, <5 piglets had levels above the lower detection limit and the bile acid was therefore excluded; T‐, taurine conjugated.

### 3.7 Gene expression

**FIGURE 6:**
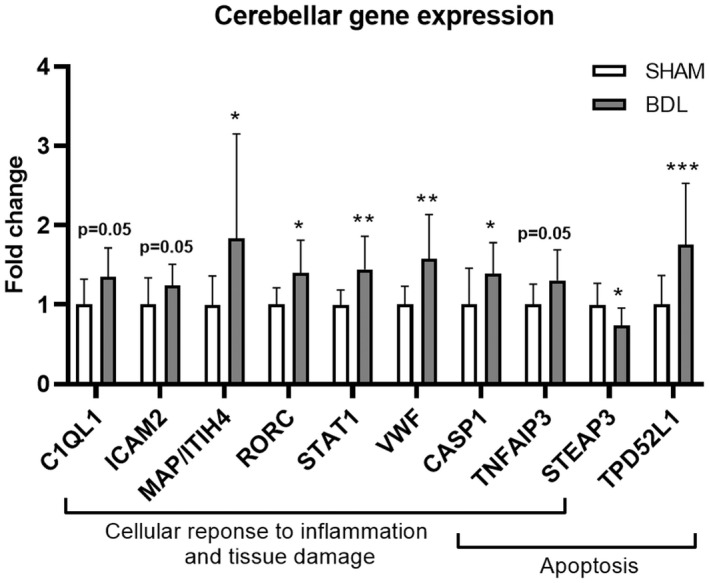
Relative qPCR gene expression in the cerebellum of SHAM‐operated (SHAM, *n* = 11) and bile duct‐ligated (BDL, *n* = 11) piglets on day 26. Statistical analysis was performed on the absolute data. *p ≤ 0.05, **p < 0.01, ***p < 0.001.

RNA sequencing only identified one gene, *TPD52L1*, encoding an apoptosis‐related tumor protein D53, that was increased in the BDL relative to SHAM group (*p* < 0.05; data not shown). The expression of *TPD52L1* was verified by qPCR (*p* < 0.001; Figure 6). Furthermore, qPCR analysis identified other apoptosis‐related genes to be up‐ (*CASP1*, *TNFAIP3*) or downregulated (*STEAP3*) in the BDL relative to SHAM group (all *p* ≤ 0.05; Figure 6). Additionally, genes related to the cellular response to inflammation and tissue damage i.e., *CASP1*, *C1QL1, ICAM2, MAP/ITIH4*, *RORC*, *STAT1*, *TNFAIP3* and *VWF* were also upregulated in the BDL group (all *p* ≤ 0.05; Figure 6). There were no differences between groups for the remaining analyzed genes (Table S8).

## 4 DISCUSSION

In this study, we report that bile duct ligation induces significant molecular changes in the brain of neonatal piglets including (i) a reduction in total lipids, including PI and PS and increases in lysophospholipids, (ii) a switch toward a more conjugated and hydrophilic bile acid profile, and (iii) that these changes occur in conjunction with sensory‐motor deficits, primarily balance.

The BDL piglet model displayed increases in liver functional enzymes, clinical symptoms and histopathological changes consistent with obstructive NC in children (Feldman & Sokol, 2013; Lee & Looi, 2009). Examining motor function revealed that BDL piglets had significantly longer stance times in the gait analysis and more missteps in the balance beam test indicating that balance was compromised by cholestasis. Previous reports of motor function have mainly been limited to open field testing of adult rat models of chronic liver disease with few studies reporting on compromised coordination and balance (Dhanda & Sandhir, 2015). Such findings have been suggested to result from changes in the central nervous system including disturbed dopaminergic and serotonergic neurotransmission (Dhanda & Sandhir, 2015), increased extracellular glutamate and mGluR1 activation (Cauli et al., 2006) and neuroinflammation (Rodrigo et al., 2010). Here, sensory‐motor deficits were paralleled by changes to the concentration and composition of cerebellar lipids and bile acids and enhanced expression of genes related to inflammation and tissue damage. However, we cannot rule out that these deficits are in part related to increased ammonia levels (Jover et al., 2006), abdominal distension, and lower carcass weights, which in turn could relate to reduced muscle mass, observed in BDL piglets that may affect normal movement (Ng et al., 2018). The present study also showed that SHAM piglets spent more time in the entrance zone of the open field arena at the end of the study. This could be interpreted as one of two things: that SHAM piglets are less explorative than BDL piglets, or that SHAM piglets are able to recall this area better than BDL piglets. Indeed, rats have demonstrated impairments in cognitive function following bile duct ligation (Dhanda & Sandhir, 2015). However, evaluation of the cognitive abilities of piglets was outside the scope of this study.

The brain growth spurt in the last trimester of pregnancy and early postnatal life is characterized by lipid accretion and increased susceptibility to environmental influences such as nutrition (Dobbing & Sands, 1979; Georgieff et al., 2018; Martinez, 1992; Svennerholm & Vanier, 1972). However, there is currently very limited information on how concomitant liver disease and lipid malabsorption in NC affects the brain lipidome. Chen et al. found that concentrations of 20:4 n‐6 and 22:6 n‐3 fatty acyls were reduced in the brain of BDL rats 2–4 weeks after surgery. These results had an inverse correlation with the duration of bile duct ligation, severity of liver fibrosis, and plasma bilirubin (Chen et al., 2010). Using a more comprehensive lipidomics approach, our findings suggest that the total amount of lipids in the cerebellum was reduced in the BDL group which was mainly driven by reductions in the phospholipid classes PI, PS, and PC O‐. Glycerophospholipids are essential for cellular functioning given their roles in neuronal membrane structure, dynamics, and signaling (Farooqui et al., 2000). In particular, PI‐derived phosphoinositide metabolism and signaling in the brain has been described to be more prominent in infants and young rats suggesting a role in brain development (Balduini et al., 1987; Yu et al., 2020). In the present study, we were unable to detect changes related to phosphoinositide metabolism at the gene level, although a broader analysis of phosphoinositides and their downstream signaling pathways is warranted. Interestingly, LPE and several LPC species derived from the hydrolysis of PE and PC were increased in the BDL group. While LPC has been reported to have demyelinating and endothelial barrier disrupting properties in the central nervous system of mice (Muramatsu et al., 2015; Plemel et al., 2018), the significance of elevated LPE in the brain, which has also been reported in animal models of cerebral ischemia (Sabogal‐Guáqueta et al., 2018) and traumatic brain injury (Guo et al., 2017), remain unclear.

In recent years, bile acids have been suggested to be involved in the pathogenesis of hepatic encephalopathy (DeMorrow, 2019). In this study, we demonstrated a 35‐ and 11‐fold increase in the level of total bile acids in plasma and cerebellum of BDL piglets, respectively. We also showed that the molecular bile acid profiles differed between groups with conjugated HCA dominating in the BDL group and HCAs, HDCAs and CDCAs in the SHAM group. This is different to BDL rats where LCA was the most abundant bile acid in serum and whole brain samples (Tripodi et al., 2012) and NC patients where conjugated CDCA and CA make up the majority of plasma bile acids (Zhou et al., 2015). Species‐dependent differences in the plasma bile acid profile are well known. Compared to humans, pigs have a dominance of hyo‐forms, less sulfation, and more hydroxylation of plasma bile acids (Thakare et al., 2018). However, the significance of increased total brain bile acid levels and distinct bile acid profiles that develop as a result of cholestasis warrants further study. The bile acid profile of BDL animals in this study is generally considered to be hydrophilic (Thakare et al., 2018). Zhang et al. demonstrated that pathological levels of hydrophilic bile acids in cholestatic mice were unable to induce hepatocellular damage ex vivo but could stimulate pro‐inflammatory mediators, thus indicating that they mainly have pro‐inflammatory rather than cytotoxic properties (Zhang et al., 2012). Similarly, hydrophilic T‐CA has been suggested to promote microglia activation and subsequent neuroinflammation via sphingosine‐1‐phosphate receptor 2 (S1PR2) signaling (McMillin et al., 2017; Pavlović et al., 2018). Here, we show upregulated expression of cerebellar genes related to a low grade cellular response to inflammation, tissue damage, and apoptosis in neonatal BDL piglets after 3 weeks, supporting the previous observation of cerebellar neuroinflammatory responses in BDL rats (Rodrigo et al., 2010). Of note, *C1QL1*, encoding complement C1q‐related factor, which was upregulated in BDL piglets, is expressed in areas of the brain related to motor function including purkinje cells of the cerebellum (Bérubé et al., 1999) and has also been shown to cause reduction of synaptic density in cultured neurons (Bolliger et al., 2011) highlighting a potential link between BDL pathology and motor function. A weakness of this study is, however, that these changes have not been correlated with changes in protein expression. More studies are needed to confirm the isolated effects of bile acids on neuronal cells.

Surprisingly, contrary to findings from a mouse model of acute liver failure‐induced hepatic encephalopathy (McMillin et al., 2016, 2017), the present study showed no differences in the cerebellar expression of genes related to bile acid signaling such as farnesoid X receptor (*FXR*), small heterodimer partner and *S1PR2* between BDL and SHAM groups. This could in part relate to our more chronic disease model. Additionally, no differences were observed in the expression of bile acid transporters like *ASBT* and *OATP1A2* which could suggest that bile acids gain access to the brain by other mechanisms under cholestatic conditions. This could include increasing blood–brain barrier permeability by disruption of tight junction proteins, as previously demonstrated in BDL rats (Dhanda & Sandhir, 2018; Quinn et al., 2014).

Hepatic production of bile acids through the classical bile acid synthesis pathway was increased by bile duct ligation as indicated by elevated C4 and reduced FGF‐19 levels in plasma in the BDL group. These results are contrary to those reported in biliary atresia patients at the time of kasai portoenterostomy (Johansson et al., 2020). One explanation for this may be related to the reported antagonistic effects of HCA species on FXR activation (Zheng et al., 2021) which could account for reduced secretion of FGF‐19 from hepatocytes and low levels of circulating FGF‐19 in BDL piglets (Johansson et al., 2020; Song et al., 2009). Targeting this pathway has recently shown promising results for prevention of liver pathology in cholestatic piglets, although the translatability to humans and implications for brain function have yet to be determined (Xiao et al., 2021).

## 5 CONCLUSION

In summary, we report that cholestasis induced by bile duct ligation in neonatal piglets causes sensory‐motor deficits and significant molecular changes in the cerebellum. Further research is needed to determine whether these findings are causally associated with the onset of NC‐associated neurodevelopmental impairments. For this purpose the NC piglet model would be beneficial.

## AUTHOR CONTRIBUTIONS

Conceptualization: Nicole Lind Henriksen, Thomas Thymann, Vibeke Brix Christensen. Methodology: Nicole Lind Henriksen, Thomas Thymann, Vibeke Brix Christensen, Stanislava Pankratova, Lars Søndergaard Johansen, Thomas Eriksen, Kerstin Skovgaard. Formal analysis: Nicole Lind Henriksen, Xiaoyu Pan. Investigation: Nicole Lind Henriksen, Matthew Domenic Lycas, Lars Søndergaard Johansen, Thomas Eriksen, Douglas G. Burrin, Svend Høime Hansen, Christer Stenby Ejsing, Richard R. Sprenger. Writing – original draft preparation: Nicole Lind Henriksen. Writing – review and editing: Nicole Lind Henriksen, Svend Høime Hansen, Matthew Domenic Lycas, Xiaoyu Pan, Thomas Eriksen, Lars Søndergaard Johansen, Richard R. Sprenger, Christer Stenby Ejsing, Douglas G. Burrin, Vibeke Brix Christensen, Thomas Thymann, Stanislava Pankratova, Kerstin Skovgaard. Visualization: Nicole Lind Henriksen. Supervision: Stanislava Pankratova, Thomas Thymann. Project administration: Nicole Lind Henriksen, Thomas Thymann, Stanislava Pankratova. Funding acquisition: Thomas Thymann, Vibeke Brix Christensen.

## FUNDING INFORMATION

This work was supported by the Danish Dairy Research Foundation and the Novo Nordisk Foundation (NNF18OC0052834).

## CONFLICT OF INTEREST

The authors declare no conflicts of interest.

## ETHICS STATEMENT

Animal experimental work was approved by The Danish Animal Experiments Inspectorate (license number 2014‐15‐0201‐00418) and reported according to ARRIVE guidelines.

## Supporting information




Figure S1.

Figure S2.

Table S1.

Video S1.
