# Supplementary material for: Cholestasis alters brain lipid and bile acid composition and compromises motor function in neonatal piglets
Source: Physiol Rep. 2022 Jul 12;10(13):e15368. doi: 10.14814/phy2.15368 (PMC9277266; doi:10.14814/phy2.15368)
Supplement: Supplementary file 1 — Figure S1. [file PHY2-10-e15368-s002.pdf]

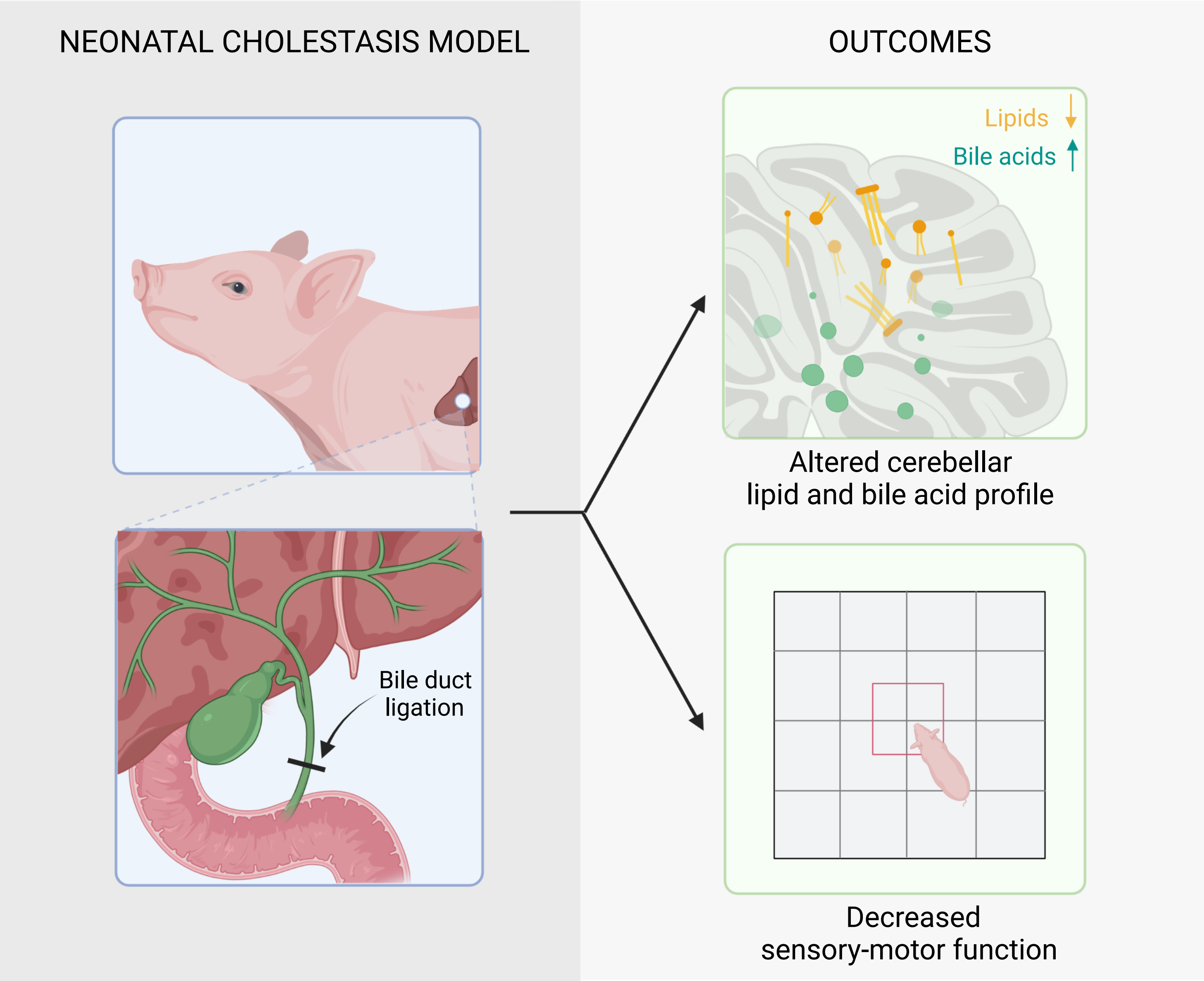
NEONATAL CHOLESTASIS MODEL
OUTCOMES
Lipids
Bile acids
Altered cerebellar lipid and bile acid profile
Bile duct ligation
Decreased sensory-motor function
