# Supplementary material for: Cholestasis alters brain lipid and bile acid composition and compromises motor function in neonatal piglets
Source: Physiol Rep. 2022 Jul 12;10(13):e15368. doi: 10.14814/phy2.15368 (PMC9277266; doi:10.14814/phy2.15368)
Supplement: Supplementary file 3 — Table S1. [file PHY2-10-e15368-s001.pdf]

Table S1: Nutrient composition of the milk formula diet given to both sham-operated (SHAM) and bile duct-ligated (BDL) piglets

**Constituents**	**Product**	**Unit**	**Diet**
Whey protein	WPI/WPC 90[1]	g/L	30
Casein	Miprodan®40[1]	g/L	30
Lactose	Variolac®936[1]	g/L	70
Vitamin/minerals	Phlexy-Vits®[2]	g/L	5
Medium chain fatty acids	Liquigen®[2]	g/L	50
Long chain fatty acids	Calogen®[2]	g/L	40
Energy		kJ	3615
Protein		g/100g	55
Carbohydrate		g/100g	59
Fat		g/100g	45

[1]Arla Food Ingredients, Viby J, Denmark

[2]Nutricia, Amsterdam, The Netherlands

Table S2: Genes and associated primer sequences used for qPCR analysis

Gene symbol	Gene name	Forward sequence (5' to 3')	Reverse sequence (5' to 3')
**Phosphatidylinositide metabolism**			
PIKC3	Phosphatidylinositol 3-Kinase Catalytic Subunit Type 3	AGTGAGAATGGTCCGAATGG	ATCACGCAATATCCAGCACA
PIKFYVE	Phosphoinositide Kinase, FYVE-Type Zinc Finger Containing	GAAGGGCTTCCAACAAACAG	ACGGTTTGTCTGTCCTCCAC
PLCB1	Phospholipase C Beta 1	CAACTGGCTGCACTGACACT	CAACTGGCTGCACTGACACT
SYNJ1	Synaptojanin 1	AAGGCGACGTGGATGACTAC	AGCTTGAAGATGGCTGGAGA
**Inflammation**			
C1QL1	Complement C1q Like 1	TTTCACCTACCACGTCCTCA	GCACCTGTCCGTTCTTGC
C3	Complement 3	ATCAAATCAGGCTCCGATGA	GGGCTTCTCTGCATTTGATG
C5	Complement 5	AAGCTGGAGAAGCCGTTGC	TTTTCGAGGTTAGCGTTCGT
CASP3	Caspase 3	CTGGCAAACCCAAACTTTTC	GTCCCACTGTCCGTCTCAAT
CD14	CD14 Molecule	AAGCTCACCGTGCTTGATCT	CCTTCCAGGGTCAGGTCAT
HMGB1	High Mobility Group Box 1	CAAGGCCCGTTATGAAAGAG	ATCTGCAGCGGTGTTATTCC
ICAM2	Intracellular Adhesion Molecule 2	GGGGTCCATACAGGACACTG	CAGCTCGTACTTCTGCGACA
IL4	Interleukin 4	GCAAACATGACCTGTTCTGTG	GCTTCAACACTTTGAGTATTTCTCC
LBP	Lipopolysaccharide binding protein	ATTCAATGTGGAGCTGTT	GGAAGCCTTCTGCCAACT
MAP/ITIH4	Major Acute Phase Protein/Inter-Alpha-Trypsin Heavy Chain 4	ATGACAGCAAGCGAACAGTG	GGGGATCCCTCTTGGTAATC
MYD88	MYD88 Innate Immune Signal Transduction Adaptor	AGCTGTAGGGGGAATGTGTG	TCAGCTGGTCTGTGGATGTG
NFKB1	Nuclear Factor Kappa B Subunit 1	CTCGCACAAGGAGACATGAA	GGGTAGCCCAGTTTTTGTCA
NLRP3	NLR Family Pyrin Domain Containing 3	GACTTTCCAGGAGTTCTTTGCTG	CCTGGTTTACAAGGCCAAAG
RORC	RAR Related Orphan Receptor C	CTAAGAGGCTCTCGGGCTTT	ATCCTGACCAGCACCACTTC
SAA	Serum Amyloid A	CAGAGATGGGCATCATTCCT	TGGCATCGCTGATCACTTTA

SELL	Selectin L	CGATATGTCAAAAACTGGACAGG	AAATGCCAACCCTGAGACTG
STAT1	Signal Transducer And Activator Of Transcription 1	CCTTGCAGAATAGAGAACATGATAC	CCTTTCTCTTGTTGTCAAGCATT
TLR4	Toll Like Receptor 4	TGGTGTCCCAGCACTTCATA	CAACTTCTGCAGGACGATGA
TNFAIP3	TNF Alpha Induced Protein 3	CCCAGCTTTCTCTCATGGAC	TTGGTTCTTCTGCCGTCTCT
VCAM1	Vascular Cell Adhesion Molecule 1	CTTGACGTGAAAGGAAGAGAAAG	GGATGCACAATAGAGCACGA
VWF	Von Willebrand Factor	CACTGAAGCGCGATGAGAC	CGTGATCCTCTTCTCCCAGA
**Oxidative stress/apoptosis**			
CASP1	Caspase 1	GAAGGACAAACCCAAGGTGA	TGGGCTTTCTTAATGGCATC
HIF1A	Hypoxia Inducible Factor 1 Subunit Alpha	TGTGTTATCTGTCGCTTTGAGTC	TTTCGCTTTCTCTGAGCATTC
NOS2	Nitric Oxide Synthase 2	GCAGCTACTGGGTCAAGGAC	GCTGTTGGTGAACTTCCACTT
OXR1	Oxidation Resistance 1	TAGACACCCCAGTGCTGATG	TGGCTCAGATGCTAATGCAC
STEAP3	STEAP3 Metalloreductase	TCAACCTCGTCATCAAGCAG	TAGATCTCCATCCGCCAGAC
TPD52L1	TPD52 Like 1	CTGGCATGATGTGCAGACTA	CTGGCATGATGTGCAGACTA
**Bile acid signalling**			
ASBT	Apical sodium bile acid transporter	ATAATGGGATGCTGTCCAGG	TAGATTAAGAGGCACAGCGG
FXR	Farnesoid X Receptor	CGAAAGAGTGGTATCTCCGATG	GCTGTAAGCAGAGCGTATTCT
GR	Glucocorticoid Receptor	TCCTGCTTTCACACGCTAAGT	ATCAGTGGGTATCAGCTCTGC
OATP1A2	Organic Anion-Transporting Polypeptide 1A2	TCGTTAGCGTCATTCTGTGC	CGTTTTGGGCAAAAAGAAAA
PXR	Pregnane X Receptor	GTCTGCTTGCAACCCATCAG	GCACTAAGAGGGCTTGGCAT
SHP	Small Heterodimer Partner	TCTTCAACCCTGATGTTCCAGG	ACAGGGCGAAAGAAGAGGTC
S1PR2	Sphingosine-1-Phosphate Receptor 2	ACCTGAGGCTCCAGAGTATTTG	TGCTGGGGCTTAGGTATTCTG
TGR5	Takeda G-Protein Coupled Receptor 5	CTGGGGAGACAGGAAGAGGA	GCAACAGGGGTGCCTGC
VDR	Vitamin D Receptor	AGCCAGCACTTCCTTACCTG	TGAAGAAGCCTTTGCAGCCT
**Reference genes**			
ACTB	Actin Beta	CTACGTCGCCCTGGACTTC	GCAGCTCGTAGCTCTTCTCC

GAPDH	Glyceraldehyde-3-Phosphate Dehydrogenase	ACCCAGAAGACTGTGGATGG	AAGCAGGGATGATGTTCTGG
HPRT1	Hypoxanthine Phosphoribosyltransferase 1	ACACTGGCAAAACAATGCAA	TGCAACCTTGACCATCTTTG
RPL13A	Ribosomal Protein L13a	ATTGTGGCCAAGCAGGTACT	AATTGCCAGAAATGTTGATGC
YWHAE	Tyrosine 3-Monooxygenase/Tryptophan 5-Monooxygenase Activation Protein Epsilon	GCTGCTGGTGATGATAAGAAGG	AGTTAAGGGCCAGACCCAAT

Table S3: Plasma biochemistry values on day 5 and 26 in sham-operated (SHAM) and bile duct-ligated (BDL) piglets (means ± sd)

		Day 5		Day 26		p value	
**Parameter**		**SHAM (n=11)**	**BDL (n=11)**	**SHAM (n=11)**	**BDL (n=11)**	**p_5**	**p_{26}**
ALAT	U/L	24.7±4.52	22.5±4.99	33.6±9.88	23.0±9.58	0.4	<0.05
ASAT	U/L	36.4±4.27	37.1±6.59	50.0±23.8	128±152	0.6	<0.05
ALP	U/L	1757±432	1743±452	910±310	1455±393	0.9	<0.001
GGT	U/L	39.1±6.27	33.8±9.37	23.6±7.81	171±149	0.2	<0.001
Creatinine kinase	U/L	303±164	335±260	531±335	722±446	0.7	0.3
Creatinine	µmol/L	43.6±6.56	44.6±4.27	44.7±5.48	42.0±6.43	0.8	0.2
Total bilirubin	µmol/L	0.36±0.67	0.18±0.40	0.18±0.40	34.3±9.68	0.3	<0.001
Urea	mmol/L	2.55±1.16	2.93±1.15	2.95±0.91	2.89±0.78	0.7	0.9
Albumin	g/L	21.1±0.95	21.6±1.93	26.3±3.86	22.5±3.00	0.5	<0.01
Total protein	g/L	50.8±3.44	51.3±4.81	41.6±4.04	38.3±3.94	0.8	0.05
Iron	µmol/L	27.5±7.33	24.6±9.34	12.6±6.09	21.9±7.39	0.5	<0.01
Cholesterol	mmol/L	2.23±0.33	2.30±0.25	1.66±0.31	2.24±0.28	0.7	<0.001
Phosphate	mmol/L	2.09±0.25	1.97±0.19	2.62±0.26	2.32±0.17	0.2	<0.01
Calcium	mmol/L	2.99±0.13	2.96±0.23	2.73±0.19	2.66±0.14	0.9	0.2
Magnesium	mmol/L	0.96±0.06	0.97±0.08	0.92±0.08	0.84±0.08	0.7	<0.01
Sodium	mmol/L	140±2.75	140±4.90	140±5.43	140±1.45	0.7	0.2
Potassium	mmol/L	6.52±0.88	5.93±0.84	4.19±0.17	4.57±0.65	0.2	<0.05
Triglycerides	mmol/L	0.52±0.14	0.47±0.07	0.18±0.07	0.39±0.09	0.3	<0.001
Conjugated bilirubin	µmol/L	0.36±0.50	0.18±0.40	0.18±0.40	29.6±8.26	0.4	<0.001
Ammonium	µmol/L	162±43.2	136±60.4	47.2±12.4	66.7±20.1	0.3	<0.01

ALAT = alanine transaminase, ASAT = aspartate transaminase, ALP = alkaline phosphatase, GGT = gamma-glutamyltransferase

Table S4: Organ weights relative to body weight on day 26 in sham-operated (SHAM) and bile duct-ligated (BDL) piglets (means ± sd)

	Relative organ weight (g/kg)		
**Organ**	**SHAM (n=11)**	**BDL (n=11)**	**p value**
Small intestine full[1]	69.4±13.0	75.3±11.6	0.2
Small intestine empty	56.4±7.53	51.8±9.15	0.3
Stomach full[1]	27.6±9.06	34.3±8.06	0.08
Stomach empty	8.45±0.79	9.02±1.12	0.2
Colon full[1]	28.8±6.36	36.5±7.91	<0.05
Liver	33.9±3.26	61.7±7.90	<0.001
Spleen	5.50±1.26	5.00±1.28	0.4
Heart	7.99±1.16	7.70±1.74	0.7
Lungs	16.6±3.75	16.3±3.57	0.9
Kidneys	9.54±1.03	10.3±1.93	0.2
Carcass[2]	738±20.4	680±17.9	<0.001

[1]Piglets were fed a standardized bolus of 15 ml/kg of the formula diet 1 hour prior to euthanasia

[2]Carcass represents the whole body after removal of all internal organs. Two piglets from the BDL group have been removed due to measurement errors (BDL n=9)

Table S5: Absolute brain regional weights and water content on day 26 in sham-operated (SHAM) and bile duct-ligated (BDL) piglets (means ± sd)

Absolute brain weights (g)			
**Brain region**	**SHAM (n=11)**	**BDL (n=11)**	**p value**
Total weight	51.4±5.41	49.9±2.82	0.4
Cerebellum	5.89±0.49	5.61±0.32	0.1
Cerebrum	39.5±4.37	38.3±2.59	0.4
Brain stem[1]	5.32±0.67	5.16±0.30	0.3
Left hippocampus	0.80±0.09	0.82±0.10	0.7
Left caudate nucleus	0.42±0.08	0.40±0.05	0.2
Water percentage[2]	0.800±0.003	0.804±0.005	0.1

[1]One missing value from a piglet in the BDL group (BDL n=10)

[2]Calculated as the difference in left cerebral hemisphere weight before and after dehydration for 2 weeks at 50 degrees

Table S6: Open field test and gait analysis measurements on day 15 and 25 in sham-operated (SHAM) and bile duct-ligated (BDL) piglets (means ± sd)

		**Day 15**		**Day 25**		**p value**		
**Parameter**		**SHAM (n=11)**	**BDL (n=11)**	**SHAM (n=11)**	**BDL[2] (n=11)**	**p[1]**	**p_{15}**	**p_{25}**
Open field test								
Distance travelled	cm	2169±702	1976±467	2230±1235	2239±944	0.9	-	-
Velocity	cm/s	18.1±5.85	16.5±3.88	18.7±10.1	18.7±7.86	0.9	-	-
Time zone 1	s	22.7±11.9	23.2±10.6	22.7±24.6	27.9±16.7	0.3	-	-
Time zone 2	s	18.1±10.1	23.3±13.2	18.9±14.7	25.4±15.3	0.1	-	-
Time zone 3	s	28.4±15.0	25.8±13.4	23.0±17.6	32.5±17.2	0.5	-	-
Time center	s	56.9±25.4	49.3±14.2	54.3±25.2	51.5±17.5	0.7	-	-
Time perimeter	s	63.2±25.4	70.7±14.2	63.5±25.9	68.5±17.5	0.5	-	-
Number of crossings (center/perimeter)		6.45±4.59	5.18±4.29	5.18±3.31	4.82±2.36	0.5	-	-
Gait analysis								
Abs stride length	cm	23.0±2.79	22.5±2.88	28.9±1.71	26.7±3.69	0.1	-	-
Abs swing time	s	0.25±0.02	0.27±0.02	0.25±0.02	0.26±0.04	0.2	-	-
Speed	cm/s	18.1±6.00	16.5±4.42	25.1±5.07	20.4±8.80	0.1	-	-
Norm stride length		1.08±0.09	1.07±0.12	1.17±0.06	1.12±0.13	0.4	-	-
Norm stance time		269±81.1	275±52.5	194±32.0	258±86.3	NA	0.6	<0.05
Norm swing time		54.4±4.63	56.9±4.22	48.5±4.94	51.7±8.34	0.1	-	-

[1]Overall p value for treatment across time when there was no interaction between treatment and day

[2]BDL n=10 for gait analysis on day 25

NA = not assessed due to interaction between treatment and day

Abs = absolute, Norm = normalized to piglet shoulder height

Table S7: Cerebellar lipid species only detected in either the sham-operated (SHAM, n=8-11) or bile duct-ligated (BDL, n=8-11) group (means+sd)

Lipid species	SHAM	BDL	Lipid species	SHAM	BDL
	pmol/µg protein			pmol/µg protein	
Cer 18:1;2/18:1;1	ND	0.0094±0.0054	PI 32:2	0.0006±0.0003	ND
Cer 38:0;3	0.0061±0.0022	ND	PI 18:0-21:3	ND	0.0006±0.0003
CL 66:3	ND	0.0035±0.0014	PI 42:10	0.0013±0.0006	ND
CL 66:4	ND	0.0057±0.0031	PS 17:0-22:4	ND	0.0247±0.0067
CL 68:5	ND	0.0168±0.0042	PS 18:1-21:3	ND	0.0204±0.0072
CL 68:6	ND	0.0027±0.001	PS 20:3-22:6	0.2031±0.0522	ND
CL 71:4	ND	0.0044±0.0039	PS 20:4-22:5	0.0445±0.007	ND
CL 71:5	0.0036±0.0015	ND	SHexCer 46:2;2	0.0012±0.0008	ND
CL 73:8	0.0016±0.0008	ND	SM 20:0;2/18:2	0.001±0.0006	ND
CL 74:5	ND	0.0074±0.0027	SM 18:0;2/22:2	0.0058±0.0053	ND
CL 76:7	ND	0.0026±0.0013	SM 41:0;2	0.0101±0.01	ND
DAG 42:3	0.0027±0.0014	ND	SM 43:1;2	ND	0.0059±0.003
DMPE 32:1	ND	0.0012±0.0005	SM 44:1;2	0.0173±0.0097	ND
DMPE 16:0-22:6	ND	0.0015±0.0004	SM 20:1;2/24:1	0.1041±0.0598	ND
GM1 32:0;2	0.0018±0.0011	ND	TAG 48:1(FA 14:1)	ND	0.0002±0.0004
GM1 32:2;3	0.0908±0.0611	ND	TAG 49:1(FA 15:0)	ND	0.0016±0.0035
GM1 36:1;3	0.0011±0.0007	ND	TAG 49:1(FA 16:0)	ND	0.0028±0.0058
GM1 40:0;2	0.0049±0.003	ND	TAG 49:1(FA 16:1)	ND	0.0021±0.0045
GM1 40:2;2	0.0031±0.0026	ND	TAG 49:1(FA 17:1)	ND	0.0009±0.0019
HexCer 18:1;2/20:1;1	ND	0.0027±0.002	TAG 49:1(FA 18:1)	ND	0.0019±0.0041
HexCer 45:1;3	0.0054±0.0038	ND	TAG 50:0(FA 15:0)	ND	0.0002±0.0003
LPC 17:2	ND	0.0016±0.001	TAG 50:1(FA 15:0)	ND	0.0004±0.0007
LPC 19:1	ND	0.0017±0.0016	TAG 50:1(FA 17:1)	ND	0.0004±0.0006
LPC 21:2	ND	0.001±0.0007	TAG 50:2(FA 17:0)	ND	0.0003±0.0007
LPC 22:3	ND	0.0005±0.0002	TAG 50:3(FA 14:1)	ND	0.0002±0.0005
LPC O-16:0	0.0006±0.0004	ND	TAG 52:0(FA 14:0)	ND	0.0001±0.0003
LPC O-18:2	0.0004±0.0002	ND	TAG 52:1(FA 14:0)	ND	0.0001±0.0002
LPC O-20:3	0.0004±0.0002	ND	TAG 52:3(FA 14:0)	ND	0.0001±0.0002
LPE 17:1	ND	0.0021±0.0018	TAG 54:2(FA 20:0)	0.0001±0.0001	ND
LPE 21:3	ND	0.0012±0.0008	TAG 54:2(FA 22:1)	0.0001±0.0001	ND

LPI 20:3	0.0024±0.0006	ND	TAG 54:3(FA 22:3)	ND	0.0001±0.0001
LPS 16:1	0.0003±0.0001	ND	TAG 56:5(FA 20:2)	ND	0.0001±0
LPS 20:0	ND	0.0003±0.0002	TAG 56:7(FA 20:5)	ND	0.0001±0.0001
LPS 20:2	ND	0.0006±0.0004	TAG 58:2(FA 18:0)	0.0002±0.0001	ND
MLCL 52:2	ND	0.0029±0.0021	TAG 58:2(FA 18:1)	0.0015±0.0009	ND
MLCL 52:3	ND	0.0131±0.009	TAG 58:2(FA 22:0)	0.0007±0.0004	ND
MLCL 56:8	0.0024±0.0012	ND	TAG 58:2(FA 22:1)	0.0028±0.0029	ND
MLCL 58:8	ND	0.0043±0.0027	TAG 58:6(FA 16:0)	0.0001±0.0001	ND
NEFA 19:4	ND	0.0009±0.0005	TAG 58:6(FA 18:0)	0.001±0.0006	ND
PA 18:0-22:5	ND	0.0086±0.0054	TAG 58:6(FA 18:1)	0.0003±0.0002	ND
PC 40:9	0.0718±0.0139	ND	TAG 58:6(FA 20:4)	0.0001±0.0001	ND
PC O-31:0	ND	0.0034±0.0023	TAG 58:6(FA 22:4)	0.0001±0.0001	ND
PC O-42:9	ND	0.0036±0.0019	TAG 58:6(FA 22:5)	0.0003±0.0002	ND
PE 30:0	ND	0.0016±0.0008	TAG 58:6(FA 22:6)	0.0013±0.0009	ND
PE 22:2-22:6	0.0216±0.0069	ND	TAG 58:8(FA 16:0)	0.0001±0.0001	ND
PE 22:6-22:6	0.0638±0.0488	ND	TAG 58:8(FA 22:4)	0.0001±0.0001	ND
PE O-40:0	0.0131±0.0076	ND	TAG 58:8(FA 22:5)	0.0001±0.0001	ND

ND = not detected – below lower limit of detection

Table S8: qPCR gene expression in cerebellum tissue on day 26 in sham-operated (SHAM) and bile duct-ligated (BDL) piglets (means+sd).

	Relative gene expression		
**Gene**	**SHAM (n=8-11)**	**BDL (n=7-11)**	**p value**
**Phosphoinositide metabolism**			
PIKC3	1.00±0.12	1.12±0.22	0.3
PIKFYVE	1.00±0.15	1.15±0.30	0.5
PLCB1	1.00±0.09	1.13±0.24	0.2
SYNJ1	1.00±0.18	1.21±0.29	0.1
**Inflammation**			
C3	1.00±0.48	1.30±0.42	0.09
C5	1.00±0.25	1.10±0.33	0.4
CASP3	1.00±0.14	1.07±0.13	0.2
CD14	1.00±0.38	1.19±0.38	0.2
HMGB1	1.00±0.29	1.16±0.20	0.1
IL4	1.00±0.37	1.18±0.71	0.6
LBP	1.00±0.39	1.02±0.41	0.9
MYD88	1.00±0.24	0.89±0.17	0.4
NFKB1	1.00±0.13	1.08±0.14	0.3
NLRP3	1.00±0.42	1.23±0.30	0.1
SAA	1.00±1.65	1.30±1.28	0.2
SELL	1.00±0.25	1.31±0.65	0.3
TLR4	1.00±0.38	1.03±0.51	0.8
VCAM1	1.00±0.16	1.07±0.21	0.5
**Oxidative stress/apoptosis**			
HIF1A	1.00±0.18	1.13±0.28	0.3
NOS2	1.00±0.45	1.03±0.47	0.4
OXR1	1.00±0.09	1.05±0.12	0.4
**Bile acid signalling**			
ASBT	1.00±0.25	1.02±0.33	0.2
FXR	1.00±0.42	1.07±0.50	0.5
GR	1.00±0.28	1.09±0.18	0.5
OATP1A2	1.00±0.49	1.34±0.59	0.2
PXR	1.00±0.51	1.24±0.30	0.3
SHP	1.00±0.28	1.04±0.35	0.9

S1PR2	1.00±0.50	1.25±0.77	0.8
TGR5	1.00±0.40	0.85±0.15	0.3
VDR	1.00±0.62	0.87±0.31	0.7

Video S1: Video recordings from the balance beam test including tracking of snout, front shoulder, tail base and each foot for the gait analysis. The videos show an example of a clear run with no missteps (A) and a piglet stepping beside the beam (B).
